# Diagnostic utility of trigeminocervical reflex and quantitative sensory testing in horses with trigeminal-mediated headshaking

**DOI:** 10.1093/jvimsj/aalag199

**Published:** 2026-09-01

**Authors:** Melanie J Käfer-Karrer, Claudia Spadavecchia, Mathieu de Preux, Lucia Unger

**Affiliations:** Swiss Institute of Equine Medicine (ISME), Department of Clinical Veterinary Medicine, Vetsuisse Faculty, University of Bern, 3001 Bern, Switzerland; Section of Anesthesiology and Pain Therapy, Department of Clinical Veterinary Medicine, Vetsuisse Faculty, University of Bern, 3001 Bern, Switzerland; Swiss Institute of Equine Medicine (ISME), Department of Clinical Veterinary Medicine, Vetsuisse Faculty, University of Bern, 3001 Bern, Switzerland; Swiss Institute of Equine Medicine (ISME), Department of Clinical Veterinary Medicine, Vetsuisse Faculty, University of Bern, 3001 Bern, Switzerland

**Keywords:** electromyography, quantitative sensory testing, secondary headshaking, trigeminal-mediated headshaking, trigeminocervical reflex

## Abstract

**Background:**

Assessment of trigeminal nerve (TN) function in horses with trigeminal-mediated headshaking (TMHS) using trigeminocervical reflex (TCR) and quantitative sensory testing (QST) has not been investigated.

**Hypothesis/Objectives:**

Horses with TMHS show lower TCR and QST thresholds than controls. A subset shows left-to-right differences.

**Animals:**

Privately owned horses with TMHS (*n* = 12) and secondary headshaking (sHS; *n* = 4), from a referral hospital. Headshaking-free controls (*n* = 14), from the hospital’s teaching herd or privately owned.

**Methods:**

Prospective case–control-study. In unsedated horses, TCR was assessed using an automated threshold-tracking device via infraorbital nerve stimulation with concurrent splenic muscle electromyography. QST included evaluation of mechanical nociceptive, tactile sensory, and thermal nociceptive thresholds, assessed bilaterally at 2 sites. Group comparisons used t-tests or Mann–Whitney U tests. Univariate logistic regression assessed TCR and QST as predictors of TMHS.

**Results:**

Horses with TMHS had significantly lower TCR thresholds than controls (0.82 ± 0.22 mA vs. 1.16 ± 0.30 mA, Hedges’ g = 1.24, 95% CI, 0.41-2.06, *t* = −3.31, *P* = .003), and logistic regression predicting TMHS showed good performance (*R*^2^ of 0.73, area under the curve of 0.83). No significant left-to-right differences were identified. In sHS horses, lower thresholds were observed on the affected side. QST revealed no significant differences between groups.

**Conclusions and clinical importance:**

Lower TCR thresholds support altered TN function in TMHS. TCR might be a useful adjunct diagnostic tool, whereas QST did not identify affected horses, reflecting heterogeneous somatosensory phenotypes or limited sensitivity of the QST protocol.

## Abstract Translated to German

**Zusammenfassung:**

**Hintergrund**

Die Beurteilung der Funktion des Nervus trigeminus bei Pferden mit Trigeminus-mediiertem Headshaking (TMHS) mittels trigeminocervicalem Reflex (TCR) und quantitativer sensorischer Testverfahren (QST) wurde bisher nicht systematisch untersucht.

**Hypothese**

Pferde mit TMHS weisen niedrigere TCR- und QST-Schwellenwerte auf als Kontrollpferde. Ein Teil der Pferde zeigt eine Links-Rechts-Differenz.

**Tiere**

Pferde mit TMHS (*n* = 12) und sekundärem Headshaking (sHS; *n* = 4), in Privatbesitz, vorgestellt in einer Überweisungsklinik. Kontrollpferde ohne Headshaking (*n* = 14), rekrutiert aus dem Lehrbestand der Klinik oder in Privatbesitz.

**Methoden**

Prospektive Fall-Kontroll-Studie. An unsedierten Pferden wurde der TCR mittels eines automatisierten Gerätes zur Schwellenwertbestimmung durch Stimulation des Nervus infraorbitalis bei gleichzeitiger Elektromyographie des Musculus splenius beurteilt. QST umfasste die Bestimmung mechanischer nozizeptiver, taktiler sensorischer und thermischer nozizeptiver Schwellen, jeweils bilateral an zwei Stellen. Gruppenvergleiche erfolgten mittels t-Test für unabhängige Stichproben oder Mann–Whitney-U-Test. Eine logistische Regression wurde verwendet, um TCR und QST als Prädiktoren für TMHS zu bewerten.

**Ergebnisse**

Die TCR-Schwelle war bei TMHS signifikant niedriger als bei Kontrollen (0.82 ± 0.22 mA vs. 1.16 ± 0.30 mA, Hedges’ g = 1.24, 95% CI: 0.41—2.06, t = -3.31, *P* = .003). TCR sagte das Vorliegen von TMHS mit guter Genauigkeit voraus (R^2^ = 0.73; AUC = 0.83). Es gab keine signifikante Links-Rechts-Differenz. Deskriptiv wiesen Pferde mit sHS niedrigere Schwellenwerte auf der betroffenen Seite auf. QST wies keine signifikanten Unterschiede zwischen den Gruppen auf.

**Schlussfolgerungen/klinische bedeutung**

Erniedrigte TCR-Schwellen sprechen für eine veränderte Funktion des Nervus trigeminus bei TMHS. Der TCR könnte ein nützliches ergänzendes diagnostisches Verfahren darstellen. Mittels QST konnten betroffene Pferde nicht identifiziert werden, was möglicherweise auf heterogene somatosensorische Phänotypen hinweist oder auf eine limitierte Sensitivität des QST-Protokolls zurückzuführen ist.

## Introduction

Headshaking is a disorder in horses characterized by involuntary movements of the head and neck.^1^^,^^2^ Cases with identifiable lesions are termed secondary headshaking (sHS),^3^ whereas in cases without clinically relevant disease, a primary disorder of the trigeminal nerve (TN) is suspected (trigeminal-mediated headshaking [TMHS]).^2^ Horses with TMHS often exhibit signs of nasal irritation and can display pain-related facial expressions even between overt headshaking episodes, supporting the impression of the painful nature of the disease.^4^ TMHS shows clinical similarities to human trigeminal neuralgia, a neuropathic pain condition characterized by recurrent paroxysmal facial pain.^5^ Human trigeminal neuralgia typically presents as a unilateral disorder and is associated with focal demyelination at the trigeminal root entry zone.^6^ In contrast, histological analyses have not identified structural TN abnormalities in horses with TMHS, supporting a functional rather than structural disorder.^7^^,^^8^ Although TMHS is generally considered a bilateral disorder, increased TN asymmetry has recently been reported on MRI.^9^ While the clinical relevance remains unclear, asymmetric trigeminal involvement cannot currently be excluded.

Trigeminal nerve hyperexcitability has been shown by somatosensory evoked potential (SEP) studies.^8^^,^^10^ Because SEP measurements require general anesthesia, clinical applicability as a diagnostic method is limited.

The trigeminocervical reflex (TCR) is a nociceptive reflex that can be elicited by electrical stimulation of trigeminal afferents and recorded via electromyography from cervical muscles involved in head retraction.^11^ The TCR has been characterized in humans, cats, and healthy horses^11–13^ and is used in humans to investigate central nervous lesions, cluster headache, and trigeminal neuralgia.^14–16^

Quantitative sensory testing (QST) uses controlled mechanical, tactile, and thermal stimuli to assess somatosensory function and is widely used in humans to characterize neurological and pain disorders.^17^ Patients with trigeminal neuralgia exhibit heterogeneous somatosensory phenotypes, including sensory loss (hypoesthesia) or gain (hyperalgesia).^18^ In horses, QST has mainly been investigated in healthy horses and for the assessment of osteoarthritic pain.^19–22^

A recent case report demonstrated a lowered TCR threshold and signs of tactile hypoesthesia in a horse with TMHS, highlighting the need for systematic investigation.^23^

The aim of this study was to evaluate TCR and QST as diagnostic tools to assess TN function in horses with TMHS.

We hypothesized that horses with TMHS would show lower thresholds for TCR and QST and a stronger behavioral response compared to control horses. For TCR, we hypothesized that a subset of TMHS horses would show left-to-right threshold differences.

## Materials and methods

### Animals

The study was conducted as a prospective case–control-study. Cases presented to the ISME Bern between January and November 2025 were included if a presumptive diagnosis of TMHS was made based on a standardized investigation (Protocol S1). Inclusion criteria are defined in Table 1. Horses with lesions indicative of sHS were excluded from the case cohort but were separately described.

**Table 1:** Inclusion criteria for TMHS and control groups.

TMHS group	Control group
Age 4-20 years old	Age 4-20 years old
Any sex	Any sex
Any breed	Breeds matched to TMHS group if possible
Presumptive diagnosis of TMHS based on standardized investigation (Protocol S1)	Exclusion of headshaking signs by clinical examination (Protocol S2)
Currently symptomatic with a headshaking severity of Talbot Score 2-3/3^26^	No history of headshaking-related behavior
No current treatment for headshaking except for symptomatic measures (ie, nose net)	

Control horses without headshaking signs (Protocol S2) were recruited from the ISME teaching herd or privately owned horses. Breeds were matched to cases whenever possible.

### Trigeminocervical reflex

Tests were conducted bilaterally in a fixed sequence. The first side was chosen at random. Horses were not sedated. All tests were performed by one examiner not blinded to group allocation. A feasibility score (FS; 0-5) assessed testing feasibility based on the horse’s cooperation, with higher scores indicating increasing difficulty.^24^

**Figure 1:**
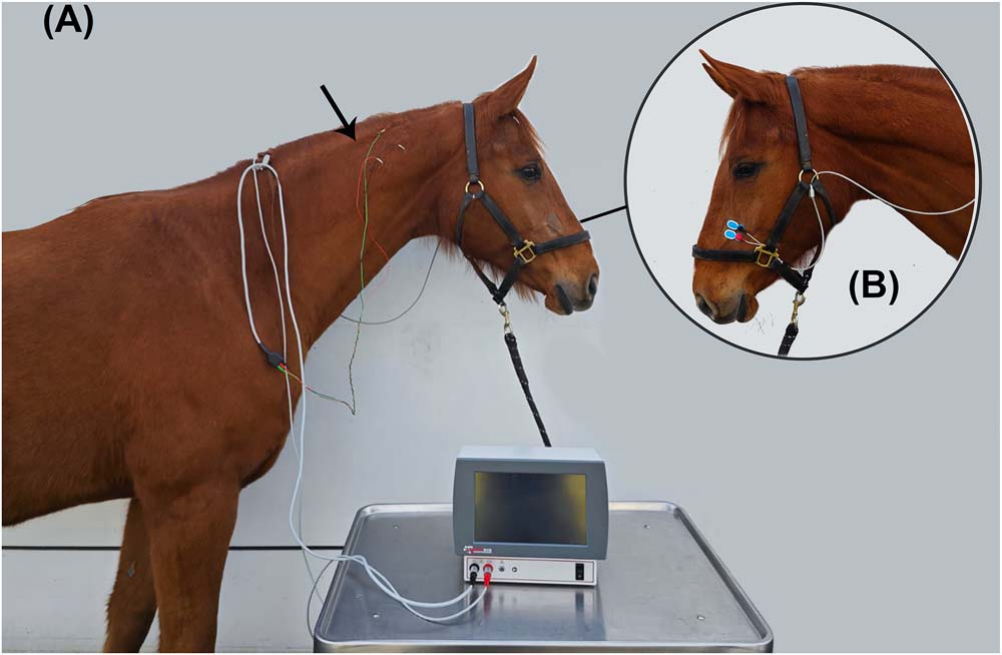
Set up for trigeminocervical reflex testing. (A) View from the right lateral side. Two subdermal recording needle electrodes are inserted subcutaneously over the splenic muscle for EMG recording (arrow), with a ground electrode placed dorsally under the mane. All electrodes are connected to the automated threshold-tracking device. (B) Simultaneous view from the left lateral side. Two self-adhesive surface electrodes are placed over the left infraorbital foramen for stimulation of the infraorbital nerve, with the cathode placed directly over the foramen and the anode rostral to the cathode.

An automated threshold-tracking device (Dolosys, Berlin, Germany) was used for TCR testing (Protocol S3).^23^ Electrical stimulation of the infraorbital nerve (ION) was performed using surface electrodes (Blue Sensor NF; Ambu, Ballerup, Denmark) placed over the infraorbital foramen (IOF). In 1 horse with a pathology within the sensory distribution of the mental nerve (MN), TCR was additionally evoked by MN stimulation as an exploratory assessment. For EMG recording, 2 needle electrodes (Spes Medica, Genova, Italy) were inserted subcutaneously over the splenic muscle (SP; Figure 1). Stimulation intensity was increased in steps of 0.1 mA until a behavioral reaction and EMG burst activity were observed. The response was confirmed by repeated stimulation at the same intensity, which was defined as the threshold. The lower threshold measured on either side was used for analysis.

Behavioral responses to stimulation were scored using a reaction score (RS; 0-5), reflecting the intensity of signs elicited by stimulation.^11^

### Quantitative sensory testing

**Figure 2:**
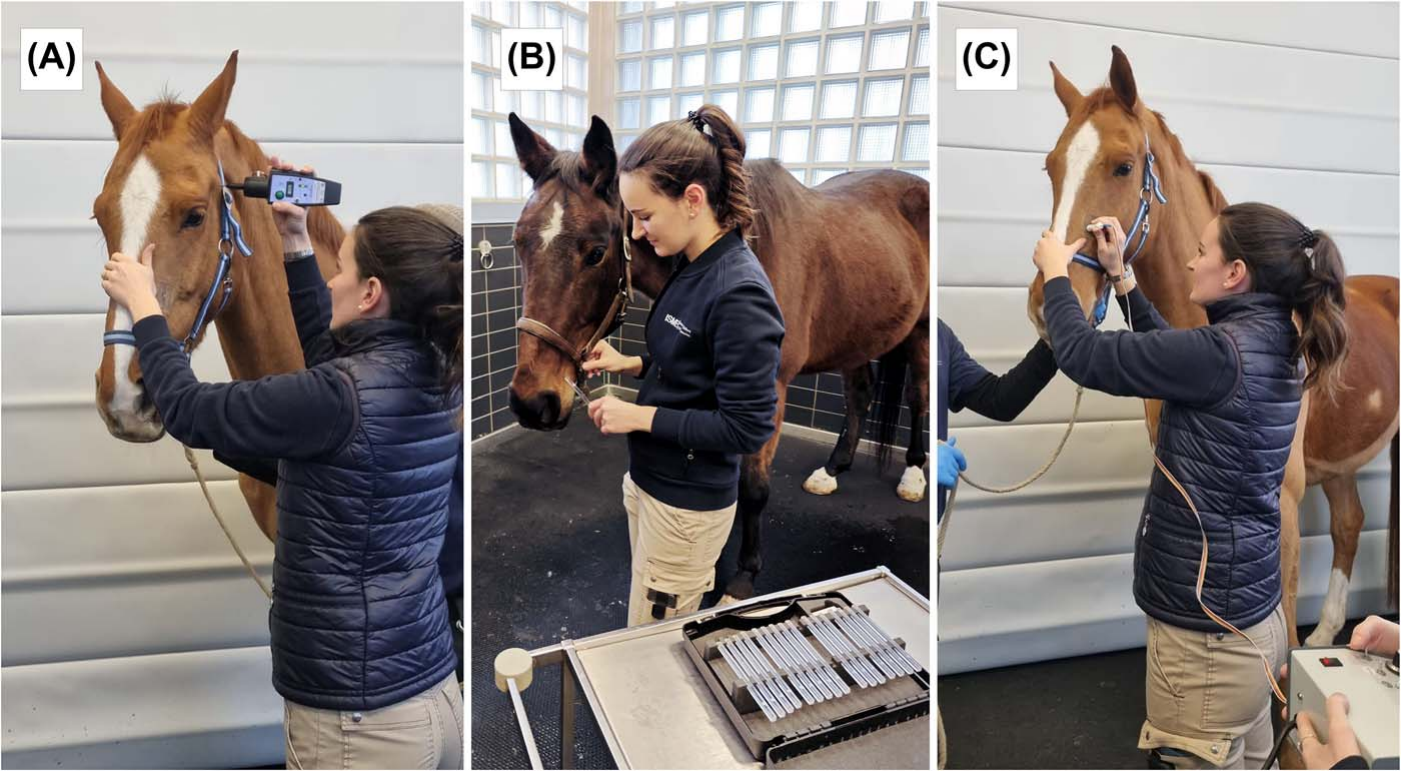
Quantitative sensory testing (QST) modalities. (A) Mechanical nociceptive threshold (MNT) measured using a handheld pressure algometer. (B) Tactile sensory threshold (TST) determined at the nostril with von Frey filaments. (C) Thermal nociceptive threshold (TNT) evaluated using a handheld thermode device.

**Figure 3:**
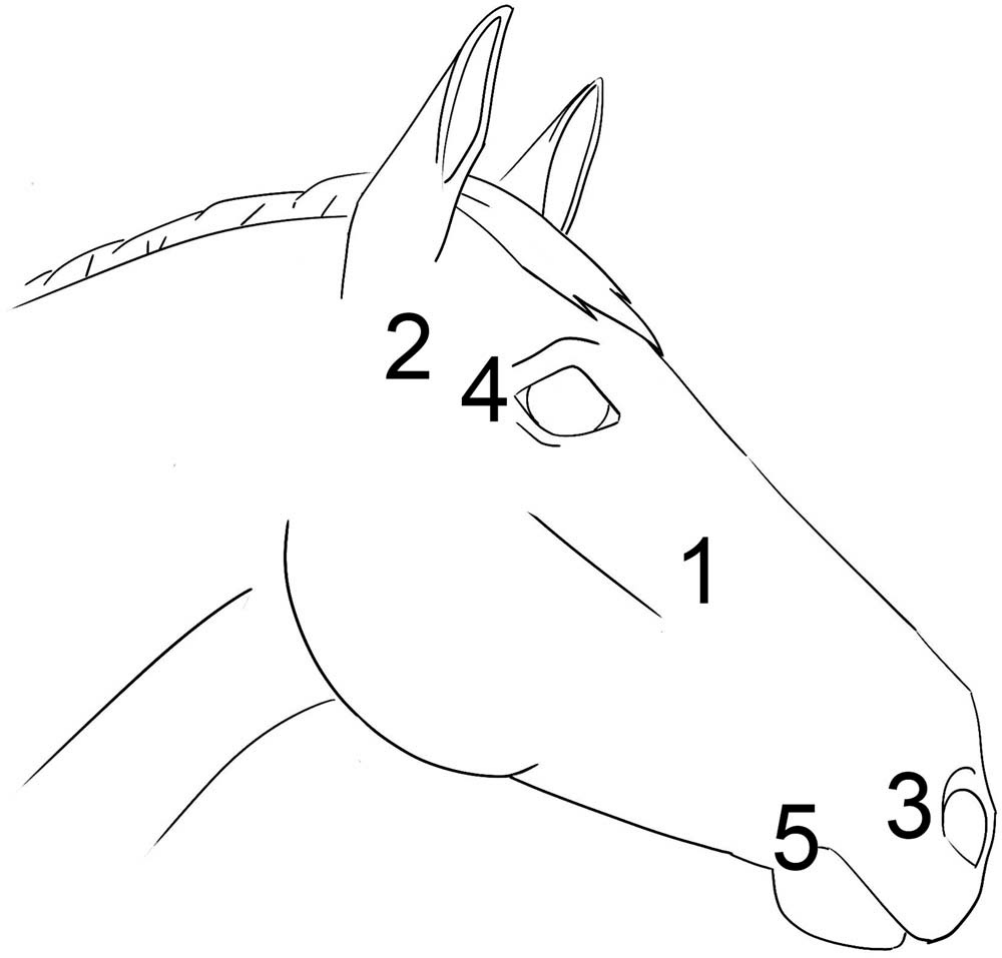
Sites for quantitative sensory testing and modalities tested at the specific site—Mechanical nociceptive (MNT), tactile sensory (TST), and thermal nociceptive (TNT) thresholds. (1) infraorbital foramen (MNT & TNT), (2) temporomandibular joint (MNT), (3) nostril (TST), (4) lateral canthus of the eye (TST), (5) mental foramen (TNT).

Mechanical nociceptive (MNT), tactile sensory (TST), and thermal nociceptive (TNT) thresholds were evaluated, as previously described (Protocol S4, Figure 2).^23^ Each test was performed at 2 sites (Figure 3). The mean of the thresholds from both sides at each site was analyzed.

### Statistical analysis

Analyses were performed using NCSS statistical software (2025; NCSS, Kaysville, USA). Group comparisons were performed using a 2-sample *t-*test for normally distributed data or a Mann–Whitney U test for non-normally distributed data. Univariate and multivariable logistic regression was used to investigate the predictive value of TCR and QST for TMHS status. Statistical significance was set at *P* < .05.

## Results

### Animals

**Figure 4:**
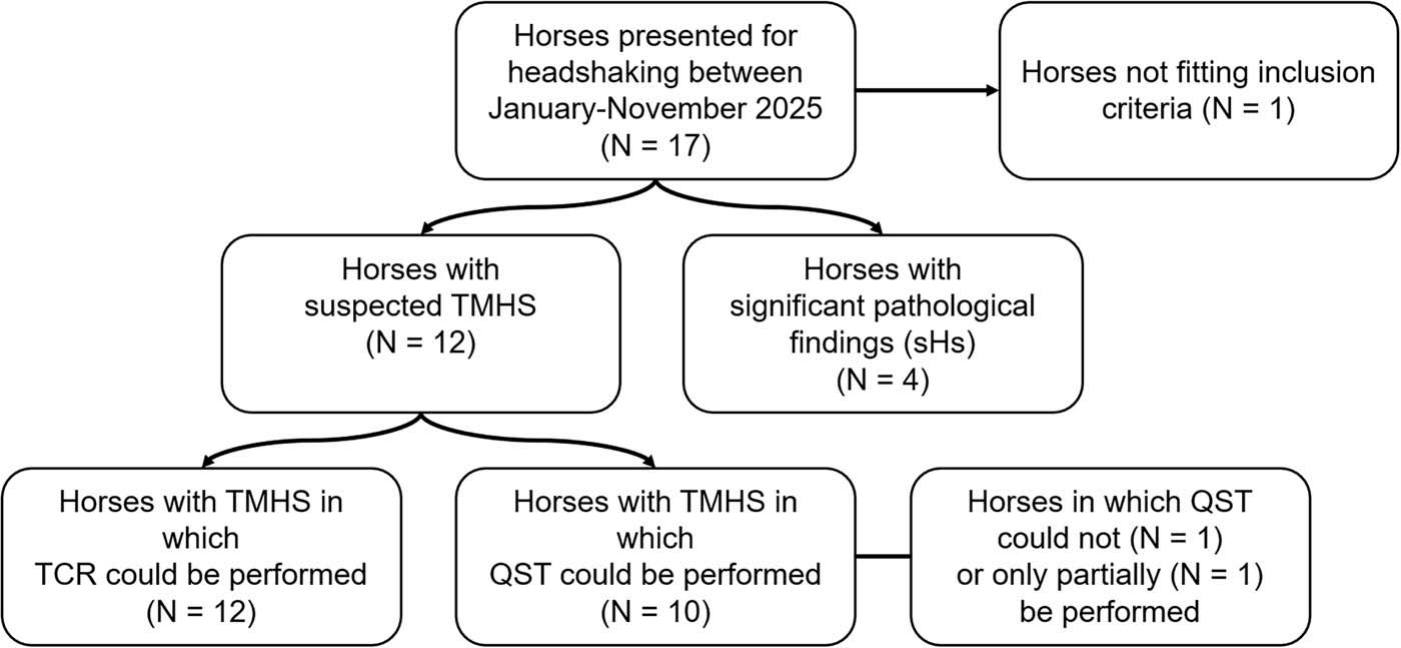
Flowchart illustrating the recruitment process of horses with trigeminal-mediated headshaking. Abbreviations: QST = quantitative sensory testing; sHS = secondary headshaking; TCR = trigeminocervical reflex; TMHS = trigeminal-mediated headshaking.

Seventeen horses were evaluated for headshaking during the study period. Twelve fulfilled the inclusion criteria and were classified as TMHS, whereas 4 were diagnosed with sHS. One horse was excluded because the headshaking severity did not reach a Talbot score ≥ 2 during the study period (Figure 4). Cases with TMHS had a median age of 12 years (IQR 8.75-15; range 6-18). Duration of clinical signs ranged from 1 month to 7 years, with a median of 12 months (IQR 8.25-14.25). Of the TMHS horses, 8 were geldings and 4 were mares. Most horses were warmbloods (7). Other breeds included 1 Freiberger, 1 Connemara, 1 Haflinger, 1 Quarter Horse, and 1 French Pony. Horses were predominantly used for leisure (6) or showjumping (4), while 1 horse was used for dressage and 1 for driving. Details on the signalment, including headshaking scores for all cases and individual diagnoses for the sHS group, are summarized in Table S5.

The control group consisted of 14 healthy horses with a median age of 11.5 years (IQR 8.5-15.75; range 5-17). Sex was equally distributed (7 geldings, 7 mares). Breed distribution was similar to the TMHS group, with warmblood being the predominant breed (8), followed by Freiberger (3), Connemara, Haflinger, and Quarter Horse (1 each; Table S6).

### Trigeminocervical reflex

Trigeminocervical reflex threshold was successfully determined in all 12 TMHS cases and 14 controls. Feasibility scores did not differ between groups (median FS: TMHS 1.5 [IQR 1.0-2.1; range 0-2.5] vs controls 1.0 [IQR 0-1.5; range 0-2.5]; U = 57; *P* = .16). No horse in either group had a FS > 3.

The mean TCR threshold was significantly lower in TMHS cases (0.82 ± 0.22 mA) than in controls (1.16 ± 0.30 mA; *t* = −3.31; *P* = .003), corresponding to a standardized effect size of g = 1.24, 95% CI, 0.41-2.06. Mean left-to-right differences did not differ significantly (TMHS 0.28 ± 0.36 mA vs. controls 0.34 ± 0.32 mA; *t* = −0.44; *P* = .66).

Cases with TMHS had a higher median RS (3.0 [IQR 2.0-3.0; range 2.0-4.0]) compared with controls (2.0 [IQR 1.3-2.0; range 1-3]; U = 36; *P* = .01), with a large effect size (rank-biserial correlation = 0.57).

**Figure 5:**
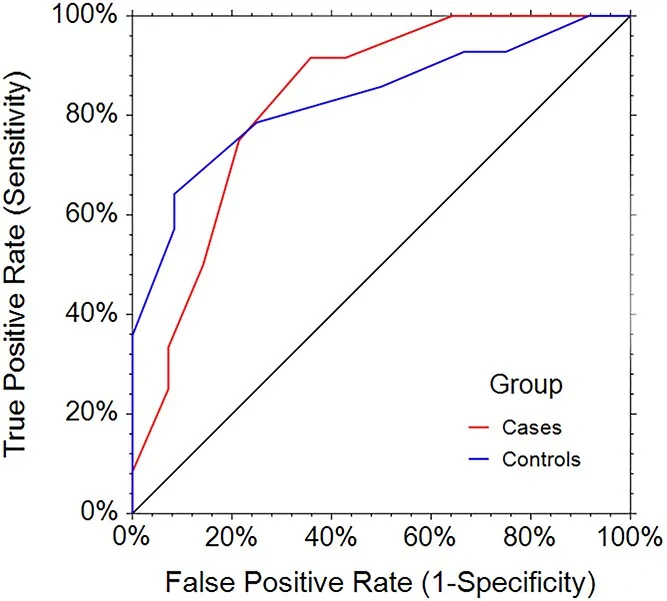
Receiver operating characteristic (ROC) curve showing the performance of the trigeminocervical reflex threshold in diagnosing trigeminal-mediated headshaking. R^2^ = 0.73; area under the curve (AUC) = 0.83.

A logistic regression with TCR threshold as predictor for TMHS yielded an *R*^2^ of 0.73 and an Area under the Receiver Operating Characteristic (ROC) Curve (AUC) of 0.83 (95% CI, 0.58-0.94; Figure 5). A probability cut-off of 0.5 (corresponding to a TCR threshold of 1.1 mA) resulted in a sensitivity of 75% (95% CI, 43-94) and a specificity of 78.6% (95% CI, 49-95).

In the group of horses affected by sHS, TCR testing could not be performed safely in one horse (Horse 13), as the horse did not tolerate being touched in the region of the IOF and became dangerous to handle. Hence, testing was aborted. Results for the remaining 3 horses (Horses 6, 10, and 17) are presented in Table S7. Descriptively, all horses showed a lower TCR threshold on the side where the pathology was located compared to the other side. In Horse 17, which was diagnosed with a mandibular cementoma, MN stimulation produced responses similar to ION stimulation, but slightly higher thresholds were recorded. Both nerves showed lower thresholds on the affected side compared to the contralateral side.

### Quantitative sensory testing

Quantitative sensory testing could be performed in all control horses and 10 of 12 TMHS cases (Figure 4). One case (Horse 7) cooperated well during mechanical and tactile stimulation but reacted to thermal stimulation with severe paroxysmal headshaking lasting for several minutes. Another case (Horse 12) was so sensitive to being touched at the head that obtaining reliable measurements was impossible. Feasibility scores were similar across both the TMHS and control groups (median FS: TMHS 2.0 [IQR 1.8-2.1; range 0-4] vs controls 1.3 [IQR 1.0-1.9; range 0-2.5]; *t* = 1.82; *P* = .08).

In 4 of 11 horses with TMHS, no response to tactile stimulation with the largest size filament used (size 18, corresponding to a calibrated weight of 50 g) was observed. At the nostril, 2 of 11 TMHS cases did not respond unilaterally and 2 of 11 did not respond bilaterally (18.2% of all tests performed), compared to 1 of 14 control horses that showed no response unilaterally (3.6%). At the lateral canthus of the eye, no reaction was recorded unilaterally in 1 of 11 TMHS cases and bilaterally in 1 of 11 (13.6%). In the control group, 1 of 14 horses did not respond unilaterally and 1 of 14 horses bilaterally (10.7%). Out of all TST tests, tactile stimulation gave a non-response rate of 15.9% in TMHS cases and 7.1% in controls.

Quantitative sensory thresholds did not significantly differ between TMHS and control horses for any modality or site (Table 2). Univariate logistic regression analyses indicated that none of the QST variables provided meaningful discriminatory value (Table 2; Figure S8).

**Table 2:** Results for quantitative sensory testing (QST).

	TMHS Cases	Controls	*t*-Value	U value	*P*-value	*R* ^2^	AUC	Sensitivity (%)	Specificity (%)
**MNT IOF**	5.7 ± 1.5 N^a^	6.5 ± 1.9 N	−1.08		.29	0.05	0.63	91.0	42.9
**MNT TMJ**	6.3 ± 2.9 N^a^	8.5 ± 3.4 N	−1.68		.11	0.10	0.66	81.8	42.8
**TST NOS**	28.33 g (0.03-80.00)^a^	7.22 g (0.03-56.65)		46	.09	0.17	0.64	45.5	92.9
**TST LCE**	24.20 g (0.06–110.00)^a^	17.98 g (0.03-110.00)		54.5	.23	0.01	0.52	36.4	64.3
**TNT IOF**	44.6 ± 2.0 °C^b^	44.6 ± 2.5 °C	−0.03		.98	0.00	0.50	50.0	50.0
**TNT MF**	46.2 ± 4.1 °C^b^	47.4 ± 2.7 °C	−0.87		.39	0.03	0.56	70.0	42.9

Abbreviations: FS = feasibility score; IOF = infraorbital foramen; LCE = lateral cantus of the eye; MF = mental foramen; MNT = mechanical nociceptive threshold; NOS = nostril; TMJ = temporomandibular joint; TNT = thermal nociceptive threshold; TST = tactile sensory threshold. Normally distributed values are given as mean ± SD and non-normally distributed data are presented as median and range. ^a^ *n* = 11. ^b^ *n* = 10.

Although a multivariable logistic regression model including all tests (TCR and all QST modalities) yielded high apparent classification performance, estimates for individual regression coefficients were unstable and confidence intervals were wide, likely reflecting the limited sample size and strong dominance of TCR. Due to these convergence issues, this multivariable model was therefore dismissed.

Results for 3 horses with sHS (Horses 6, 10, and 17) are presented in Table S7. Quantitative sensory testing was not attempted in Horse 13 due to the aversive reaction observed during TCR testing.

## Discussion

This study evaluated TCR and QST responses of horses affected by TMHS compared to control horses without signs of headshaking. We found that both methods were easy to apply and well tolerated by most horses.

The results of the present study confirm a reduced TCR threshold in horses with TMHS compared with controls as previously described in a single case report.^23^ This agrees with findings based on SEP analysis, potentially indicating hyperexcitability or hypersensitivity of the TN in TMHS.^8^^,^^10^ Although our TCR results are consistent with those reported using SEPs, the 2 techniques are not physiologically equivalent and are designed to assess different aspects of trigeminal function. To evoke the TCR, noxious stimulation must be applied to trigeminal afferents. Typically, a train-of-five 1 ms electrical pulses of sufficient (threshold) intensity is used to activate nociceptive A-δ fibers and cervical motor neurons involved in the reflex response.^11^ Thus, the TCR threshold reflects the excitability of trigeminal nociceptive reflex circuits.^25^ In contrast, SEPs are generated by repeated short electrical pulses (0.2 ms) and are primarily used to assess the integrity and conduction properties of large-fiber somatosensory pathways.^8^ In animals, reliable SEP recordings require general anesthesia, which represents a practical limitation associated with increased procedural complexity, cost, and potential risk.^8^^,^^10^

In humans, the TCR is frequently used to investigate central nervous lesions,^13^ head pain syndromes,^15^ and trigeminal neuralgia.^16^ It consists of 3 components (C1-C3), of which C3 is a long-latency polysynaptic nociceptive reflex.^25^ In healthy horses, only a single long-latency reflex corresponding to the human C3 has been identified.^11^ Due to the nociceptive nature of the C3 component, the TCR seems well suited to assess a potential dysfunction of the trigeminal system in horses with TMHS.

Enhanced behavioral responses to nociceptive trigeminal stimulation in horses with TMHS, reflected by higher RS, provide further evidence for the painful nature of the disease. In humans, a strong positive correlation has been observed between TCR variables and the intensity of neurological pain.^16^ In the present study, only horses with a Talbot Score^26^ 2-3 of 3 were included, representing moderate to severe clinical signs. Due to the small size of the study cohort, further differentiation and correlation of pain ratings (Talbot Score; History, Rest and Exercise Score^27^) with reflex threshold were not feasible.

Trigeminal neuralgia in humans is predominantly a unilateral disorder.^5^ Although the pathophysiology differs substantially between humans and horses, it remains unclear whether functional trigeminal abnormalities in TMHS are always bilateral. Previous studies have provided limited evidence regarding laterality.^7^^,^^8^ No left-to-right differences were found in SEP studies, however, sample sizes were small, or the study was not designed to assess laterality.^8^^,^^10^ In the present study using a larger study cohort when compared to Aleman et al., left-to-right differences in TCR thresholds did not differ between TMHS cases and controls. However, this does not necessarily exclude a unilateral pathophysiological process. Bilaterally abnormal TCR responses ocurr in approximately 50% of human patients with unilateral trigeminal neuralgia, likely reflecting central sensitization or altered trigeminal processing rather than bilateral peripheral disease.^16^ Given the recently reported left-to-right asymmetry in TMHS horses in TN cross-sectional area on MRI, future studies integrating functional assessment, such as TCR testing, with MRI are warranted to further investigate the potential laterality of TMHS.^9^

The lower TCR threshold (below 1 mA) on the diseased side in all horses with sHS indicates a hypersensitivity of the maxillary branch of the TN in these horses. Horses 6 and 17 both had lesions not located within the area of sensory innervation of the maxillary branch, hence a neuropathy due to direct irritation of the nerve by the pathological process seems unlikely, but this finding rather supports the theory of central sensitization in horses showing headshaking behavior. In Horse 17, the TCR was also evaluated by stimulation of the MN, a branch of the mandibular nerve. The TCR threshold for the MN was higher than for the ION but still lower on the affected side than on the contralateral. Eliciting the TCR by stimulation of the MN has not been described in the horse before. When comparing TCR responses to stimulation of different TN afferents, higher thresholds were found when stimulating the supraorbital nerve (SON) than the ION, but reflex latency and amplitude as well as visual analogue scale and numerical rating scale scores were similar for both nerves.^11^ Stimulation of other trigeminal branches for evoking a TCR has been studied in humans, however, stimulation of the SON and the MN produced more variable responses compared to the ION, which is therefore more frequently used in TCR studies.^28^ Because the TCR threshold after ION stimulation was also reduced in Horse 17, the ION appears to be a suitable site for TCR evaluation in horses with headshaking, irrespective of the location of a potential pathology. Additional cases are required to substantiate this observation.

To simplify the procedure of TCR threshold determination, EMG was recorded only from the SP muscle. In the original study describing equine TCR, EMG was also recorded from the cleidomastoideus and orbicularis oculi (OO) muscles.^11^ While the latency and duration of the reflex activity burst were similar in all the muscles, the amplitude was larger in the SP.^11^ The higher background activity observed from the SP could facilitate the reflex, and thus its recording for diagnostic purposes. Reflexive activation of the OO (blink reflex) as part of the TCR was considered while attributing the RS in the current study.

A median TCR threshold of 2.7 mA (IQR 1.9-3.5) was previously reported in healthy horses, which is higher than the mean threshold of 1.16 mA found for control horses in the present study.^11^ The threshold difference between the 2 studies might be related to different equipment used and to small but relevant methodological differences (ie, electrode size, type, and positioning, skin preparation, etc.). A similar observation has been made for measuring SEPs, highlighting the necessity to establish hospital-specific reference values before using newly established methods in clinical settings.^10^

Quantitative sensory testing provides a standardized assessment of somatosensory function in response to controlled physical stimuli and has been used in a clinical setting to quantify deficits or pain in neurological conditions in humans.^17^ In horses, few studies report the application of QST in painful conditions.^21^^,^^22^ The use of QST to characterize the somatosensory phenotype of horses with TMHS has not previously been investigated systematically.^23^ Different QST modalities assess the function of different afferent fiber populations, with tactile, mechanical, and thermal testing primarily reflecting A-β, A-δ, and C-fiber function, respectively.^29^

In the present study, no consistent pattern of QST abnormalities was identified, and none of the modalities tested provided any predictive value as a diagnostic test. Although the underlying pathophysiology in TMHS differs from that of classical human trigeminal neuralgia, similarities in functional sensory abnormalities might still exist. Human patients suffering from trigeminal neuralgia display considerable variability in QST findings, resulting in different somatosensory phenotypes.^18^^,^^30^ The most frequently observed pattern is a subclinical general hypoesthesia, which has been observed in 16.3%-47% of patients, with concurrent hyperalgesia (observed in 22% for TNT and 33% for MNT).^18^^,^^30^ If similar variability applies to horses affected by TMHS, it could explain the lack of a clear trend for the QST results observed in the present study. The non-response rate to TST testing was higher in TMHS cases (15.9%) than in controls (7.1%). Veres-Nyéki et al. also reported no response to 5.9% of the tests in a cohort of healthy horses, which is similar to our control group. While in some horses this seems to reflect an individually lower sensitivity, the higher non-response rate in TMHS horses could be indicative of lower responsiveness to tactile stimulation in a subset of horses and warrants further investigation.^21^ An assessment of the somatosensory function using QST might still be beneficial in the individual patient to monitor specific abnormalities and potential response to treatment.

All horses with headshaking included in the study were symptomatic at the time of testing. Horses with seasonal headshaking were evaluated during the period of most severe clinical signs. The behavior of QST and TCR during periods when horses are not exhibiting headshaking signs, including during remission, has not yet been evaluated. A recent study showed that horses with paroxysmal headshaking also show signs of pain between episodes of obvious headshaking.^4^ Whether trigeminal sensory abnormalities also persist during periods of clinical remission is currently unknown in horses with TMHS. In human patients with trigeminal neuralgia, no significant difference in QST parameters has been found during and outside remission.^18^ Evaluating QST and TCR in TMHS horses in remission or after targeted treatment warrants further investigation.

The study had limitations: first, the sample size was small, which could have resulted in limited statistical power. The study cohort was heterogeneous in terms of breed. Although efforts were made to match cases and controls and achieve a similar distribution between groups, the influence of breed on threshold values cannot be excluded. Breed-related differences in TCR variables have not been specifically evaluated to date. An association has not been identified before, although the small study cohort—similar to the present study—could have limited the ability to detect such effects.^11^ For QST, no differences were found in a study evaluating MNT at different locations on the distal limb between Freibergers and Warmblood horses.^24^

The influence of sex on QST has been reported in several studies in humans and horses, although the results are inconsistent. In humans, women are more sensitive than men across various QST modalities.^18^^,^^29^ In horses, no differences were identified for QST of the face.^19^ In the present study, the effect of sex was not analyzed statistically, as cases and controls were matched, and the sample size was considered insufficient to reliably assess this variable.

The diagnosis of TMHS, while based on an extensive, standardized examination protocol to rule out underlying diseases responsible for the headshaking behavior, remains only presumptive, and it is possible that some horses were falsely classified as TMHS.

Quantitative sensory testing was only evaluated at 2 sites per modality compared to 5-6 sites that had been originally described for the equine face to simplify the examination protocol and ensure the cooperation of the horses.^19^ The selected sites had previously produced the most consistent results in healthy horses.^19^ It is possible that this provided an incomplete QST assessment, especially considering that different branches of the TN were evaluated with different modalities.

Tests were always performed in the same, non-randomized order. While this might be a source of potential bias, the order was purposefully chosen this way as to not influence the TCR threshold by a possible sensitization of the ION by QST modalities, especially thermal stimulation. One horse reacted to thermal stimulation with severe paroxysmal headshaking. Thus, testing could not be continued. This justifies the use of a standardized testing order and needs to be considered when applying the tests in clinical practice.

All tests were performed by a single examiner to minimize inter-examiner variability. The examiner was not blinded to group allocation. Hence, observer bias cannot be excluded. Because all authors were involved in case recruitment, clinical examination, and experimental testing, implementation of blinding was not feasible. Although good inter- and intra-observer reliability has been reported for QST in horses,^31^ operator experience influences QST results in laboratory animals.^32^

This study presents an evaluation of TCR and QST as possible diagnostic tools for TMHS. Diagnostic accuracy of TCR appeared promising. In contrast, statistical analysis revealed no evidence of an effect of group (ie, TMHS cases versus controls) on the response to QST, which might reflect limited statistical power due to the small sample size, limited sensitivity of the QST protocol, or indicate a variation in somatosensory phenotype in TMHS. Assessment of sensory function using QST modalities might still be valuable in individual patients and could contribute to a better understanding of the pathophysiological mechanisms underlying TMHS.

## Supplementary Material

Supplementary_materials_aalag199

## Off-label antimicrobial declaration

Authors declare no off-label use of antimicrobials.

## Institutional animal care and use committee or other approval declaration

Approved by the Animal Experimentation Committee of the Canton of Bern (BE93/2024).

## Appendix—Materials and methods

### General set-up

All experimental procedures were approved by the Animal Experimentation Committee of the Canton of Bern (BE93/2024).

According to the STROBE guidelines for matched case–control studies, we used frequency (group) matching by breed to minimize potential confounding while maintaining a representative clinical population.^33^ Control cases were not specifically matched for age and sex since age and sex distributions of cases and controls were expected to be broadly comparable based on the inclusion criteria and characteristics of the clinical case population.

Horses were admitted to the clinic the day before the tests whenever possible to allow for acclimatization to the new surroundings. Testing was usually performed in the morning. No medication that could influence the results, such as sedation or analgesics, was administered on the day of the tests.

Testing was always performed in the same exam room. Care was taken to create a calm environment free of disturbances. The horse was manually restrained by a groom using a halter and rope. For every test, a second horse was present in the room to serve as a companion. Room temperature was maintained at 18-19°C.

Trigeminocervical reflex (TCR) testing was always carried out first, to exclude any influence of possible stimulation of the TN by quantitative sensory testing (QST). QST was carried out second in the following order: mechanical—tactile—thermal stimulation. All tests were performed bilaterally, with the first side chosen at random. If necessary, horses were given a 30 min break in their stall between TCR and QST.

A feasibility score (FS; 0-5) was used to assess the feasibility of testing based on the horse’s cooperation, restraint required, and confidence in the responses obtained, with higher scores indicating increasing difficulty in performing the examination.^24^

### Trigeminocervical reflex

Trigeminocervical reflex testing was performed as described previously using an automated threshold-tracking device (Dolosys, Berlin, Germany).^23^ An area of 2 × 4 cm over the IOF was clipped, shaved, and defatted with alcohol. Two self-adhesive surface stimulation electrodes (Blue Sensor NF; Ambu, Ballerup, Denmark) were placed over the IOF. The cathode was placed directly over the IOF, and the anode slightly rostral to it. If necessary, the electrodes were kept in place using a self-adhesive bandage. For EMG recording, 2 subdermal needle electrodes (Spes Medica, Genova, Italy) were inserted subcutaneously approximately 5 cm apart over the ventral aspect of the SP, parallel to the muscle fibers. A third needle electrode was placed dorsally, directly under the mane, to serve as a ground electrode. The window of interest was set at 40-200 ms post-stimulation, with 100 ms prior to stimulation used as a noise window. Recording was performed crosswise to the stimulation, ie, when stimulating the left ION, EMG was recorded from the right SP. Stimulation was always started at an intensity of 0.5 mA. Stimuli were delivered as train-of-five 1 ms pulses at 200 Hz with an interstimulus interval of 30 s ± 20%. If no clearly visible behavioral reaction could be observed, stimulation intensity was increased in steps of 0.1 mA until a clear behavioral reaction (blinking and/or retraction of the head) could be elicited and an EMG reflex in form of burst activity was present on the EMG recording. If such a reaction was observed, stimulation was repeated at the same intensity until both the behavioral reaction and EMG burst activity in the epoch of 40-200 ms post stimulation had been confirmed on at least 3 stimulations. The lowest stimulation intensity fulfilling these criteria was defined as the threshold. Stimulation intensity was then decreased to confirm that no response occurred at a lower intensity.

For stimulation of the MN in Horse 17, stimulation electrodes were placed over the mental foramen. Stimulation and recording procedures were identical to those used for the ION.

Behavioral responses to stimulation were scored using a numerical rating scale (RS; 0-5), reflecting the type and intensity of signs elicited by stimulation, such as blinking and head retraction.^11^

### Quantitative sensory testing

Quantitative sensory testing was carried out as previously described (Protocol S4).^23^ Each test was performed at 2 sites (Figure 3) that had previously been shown to produce the most consistent results in healthy horses.^19^ Testing was always performed in the same order, starting with the MNT threshold, for which a handheld pressure algometer (Prod handheld algometer, Topcat Metrology, Ely, United Kingdom) with a stimulating tip with a diameter of 2 mm was used, with a force increase of 2 N/s. The cutoff was set at 25 N. Next, the TST threshold was determined using Von Frey Filaments (SENSELab Aesthesiometer, SOMEDIC Sales, Hörby, Sweden). Only every third filament size was used (filament sizes 3-18; range 0.026-50 g). If a horse did not show any reaction to filament size 18, the result was recorded as a filament size 19 (110 g) for statistical purposes. To assess the TNT threshold, a purpose-built handheld thermode device was used. The stimulating tip had a surface area of 1 cm^2^. Temperature increase was set at a rate of 0.5-0.6°C/s and was interrupted as soon as a behavioral reaction was observed or the cutoff of 55°C was reached. If the results differed more than 1 N for the MNT or 3°C for the TNT, the test was repeated a third time, and the 2 closest values were retained for analysis.

### Statistical analysis

Analyses were performed using NCSS statistical software (2025; NCSS, Kaysville, USA). Group comparisons for TCR and QST thresholds, FS, and RS were performed using a 2-sample *t-*test for normally distributed data or a Mann–Whitney U test for non-normally distributed data. Univariate and multivariable logistic regression was used to investigate the predictive value of TCR and QST variables for TMHS status. Standardized effect sizes (Hedges’ g) were calculated from group means and pooled standard deviations with correction for small sample size. Effect sizes for Mann–Whitney U tests were calculated as rank-biserial correlations (r_rβ_), derived from the U statistic using standard formulas. Statistical significance was set at *P* < .05.

## Abbreviations

AUC: area under the curve
FS: feasibility score
IOF: infraorbital foramen
ION: infraorbital nerve
MN: mental nerve
MNT: mechanical nociceptive threshold
OO: orbicularis oculi muscle
QST: quantitative sensory testing
RS: reaction score
SEP: somatosensory evoked potential
sHS: secondary headshaking
ROC: receiver operating characteristic
SP: splenic muscle
TCR: trigeminocervical reflex
TMHS: trigeminal-mediated headshaking
TN: trigeminal nerve
TNT: thermal nociceptive threshold
TST: tactile sensory threshold
