# Supplementary material for: Diagnostic utility of trigeminocervical reflex and quantitative sensory testing in horses with trigeminal-mediated headshaking
Source: J Vet Intern Med. 2026 Sep 1;40(5):aalag199. doi: 10.1093/jvimsj/aalag199 (PMC13533209; doi:10.1093/jvimsj/aalag199)
Supplement: Supplementary file 1 [file supplementary_materials_aalag199.zip › S1_Protocol_HS.docx]

| **Examination Protocol – Headshaking** | | | | | |
| --- | --- | --- | --- | --- | --- |
|  |  |  |  | |  |
| ID Sticker |  | Talbot Score: |  |  | |
|  |  |  |  | |  |
|  |  | 0 No headshaking  1 Mild signs at exercise  2 Obvious signs at exercise but no striking or  behavioral changes  3 Most severe, with severe signs including  behavioral changes and refusal to move | | |  |

Vitals

| HR |  |
| --- | --- |
| RR |  |
| T°C |  |

Clinical examination

| General/nutritional condition |  |
| --- | --- |
| Skin/Fur |  |
| Lymph nodes |  |
| Mucosal membranes |  |
| Respiratory tract |  |
| Cardiovasculary tract |  |
| Gastrointestinal tract |  |

Ophthalmological exam

|  | Left eye | Right eye |
| --- | --- | --- |
| Eyesight |  |  |
| Menace |  |  |
| Dazzle reflex |  |  |
| Pupillary reflex |  |  |
| Eyelids |  |  |
| Conjunctiva |  |  |
| 3^rd^ eyelid |  |  |
| Sclera/cornea |  |  |
| Anterior chamber |  |  |
| Iris/pupil |  |  |
| Lens |  |  |
| Vitreous |  |  |
| Fundus |  |  |

Neurological exam

| Behavioral disorder |  |
| --- | --- |
| Posture |  |
| Mobility of the neck |  |
| Palpation of the neck |  |
| Mobility of the back |  |
| Palpation of the back |  |
| Palpebral reflex |  |
| Strabismus, Nystagmus |  |
| Symmetry of the face |  |
| Sensibility of the face |  |
| Hearing |  |
| Swallowing reflex |  |
| Tone of the tongue |  |
| Cervicofacial/-auricular reflex |  |
| Panniculus reflex |  |
| Reflex of the coronary band |  |
| Anal/perineal reflex |  |
| Tone of the tail muscle |  |
| Evaluation at walk/trot | See orthopedic exam |
| Walking with eyes covered |  |
| Walking with head elevated |  |
| Proprioceptive reflexes |  |
| Tail pull test |  |
| Walking in a circle, zig-zag |  |
| Backing up |  |
| Walking uphill/downhill |  |

Orthopedic exam

| Standing evaluation (Adspection, palpation) |  |
| --- | --- |
| Walk on a straight line |  |
| Walk in small circles |  |
| Trot on a straight line |  |
| Trot in circles |  |
| Hoof tester |  |
| Wedge test |  |
| Flexion tests |  |
| Lunging (Walk, Trot, Canter; 5min each) |  |

Oral examination

According to SVPM Zahnprotokoll.

Upper airway endoscopy

|  | Left | Right |
| --- | --- | --- |
| Nasal passage |  |  |
| Larynx/Pharynx |  |  |
| Apertura nasomaxillaris |  |  |
| Ethmoid |  |  |
| Guttural pouch |  |  |

CBCT of the skull

Scan protocol (may be adjusted depending on the size of the head):

1) Region of the incisivi and diastema (1 scan)
   a. Centered on the middle of the nose (mid-way between nostril and cranial crista facialis)
2) Region of P2-P4 (2 scans – maxilla and mandibula)
   a. Centered on cranial end of the crista facialis
3) Region of P4-M3 incl. ethmoid (2 scans – maxilla and mandibula)
   a. Cran-caud: level of the orbita
   b. Dors-ventr: level of the cranial end of the crista facialis
4) Region of the temporomandibular joints and guttural pouches (3 scans – mandibula, left and right TMJ)
   a. Cran-caud: level of the TMJ
   b. Dors-ventr: level of the cranial end of the crista facialis
5) If under GA: C1/2

Full Bloodwork (Hematology, Blood Chemistry)

Sent	☐

Performed by private vet	☐
