# Supplementary material for: Diagnostic utility of trigeminocervical reflex and quantitative sensory testing in horses with trigeminal-mediated headshaking
Source: J Vet Intern Med. 2026 Sep 1;40(5):aalag199. doi: 10.1093/jvimsj/aalag199 (PMC13533209; doi:10.1093/jvimsj/aalag199)
Supplement: Supplementary file 1 [file supplementary_materials_aalag199.zip › S3_TCR.docx]

**Trigemino-Cervical Reflex**

NOTE: always perform testing in same order (TCR – MNT – TST – TNT). First side chosen at random.

**Environment**

- Horses are to be stabled at least 4h before testing to allow for acclimatization (ideally the night before).
- Testing is always performed in the same exam room. Try to minimize disturbances (lock door, keep floor quiet).
- Personnel present: examiner, groom for handling the horse, 1-2 helpers for recording of data/technical assistance.
- Room temperature is regulated and always maintained between 18-19°C.
- A companion horse (a horse of the clinic’s teaching herd or – if available – a horse from the same stable as the patient horse) is to be present for all tests.
- Horses are given at least a 30min break in their stall between TCR and QST. Add additional breaks if needed.

**Testing**

| Dolosys Settings: | - Intensity Increase 0.1mA - Reflex Range 40ms - Reflex Range End 200ms - Randomisation 20% - Intervall 30s - No of stimuli 30 - Initial Stimulation Intensity 0.5mA |
| --- | --- |
|  |  |
| Preparation: | - Clip area approx. 2x4cm over and rostral to IOF - Shave area with plenty of betadine soap and warm water - Defat with alcohol, dry |
|  |  |

| Connect electrodes: |     - Stimulation is performed contralateral to recording site  a) 1^st^ surface electrode directly over IOF – cathode (black) 2^nd^ surface electrode rostral to 1^st^ – anode (red) b) EMG needle electrodes: green = ground – dorsally under the mane black = EMG – over splenic muscle, cranial red = EMG – over splenic muscle, caudal  - Connect electrode cables to Dolosys (black: EMG; red: STIM) - Check electric impedance (< 3 kΩ) |
| --- | --- |
|  |  |
| Stimulation: | - “Start” > stimulation will start automatically and will give one impuls every 30s +- 20% - Note time, intensity and all reactions (incl NRS score) observed; mark intensity where an EMG burst activity was first observed - Keep increasing the intensity by 0.1mA until a behavioral reaction is visible - If a behavioral reaction is observed, stay at this intensity to see whether this is repeatable (at least 3x reaction at this intensity) > afterwards decrease intensity again to see whether you can now produce a reaction at a lower intensity - *Threshold = at least 3x behavioral reaction and EMG burst activity in epoch 40-200ms post stimulation at the same intensity* |
|  |  |
|  |  |

| ID Sticker |  |  |  |
| --- | --- | --- | --- |
|  |  | Feasibility Score: |  |
|  |  |  |  |

**Trigemino-Cervical Reflex**

**1° Examined face side:	☐ left	☐ right	2° Examined face side:	☐ left	☐ right**

| **Time** | **Intensity** | **Reaction** |  | **Time** | **Intensity** | **Reaction** |
| --- | --- | --- | --- | --- | --- | --- |
|  |  |  |  |  |  |  |
|  |  |  |  |  |  |  |
|  |  |  |  |  |  |  |
|  |  |  |  |  |  |  |
|  |  |  |  |  |  |  |
|  |  |  |  |  |  |  |
|  |  |  |  |  |  |  |
|  |  |  |  |  |  |  |
|  |  |  |  |  |  |  |
|  |  |  |  |  |  |  |
|  |  |  |  |  |  |  |
|  |  |  |  |  |  |  |
|  |  |  |  |  |  |  |
|  |  |  |  |  |  |  |
|  |  |  |  |  |  |  |
|  |  |  |  |  |  |  |
|  |  |  |  |  |  |  |
|  |  |  |  |  |  |  |
|  |  |  |  |  |  |  |
|  |  |  |  |  |  |  |
|  |  |  |  |  |  |  |

* EMG reflex first present
