# Supplementary material for: Diagnostic utility of trigeminocervical reflex and quantitative sensory testing in horses with trigeminal-mediated headshaking
Source: J Vet Intern Med. 2026 Sep 1;40(5):aalag199. doi: 10.1093/jvimsj/aalag199 (PMC13533209; doi:10.1093/jvimsj/aalag199)
Supplement: Supplementary file 1 [file supplementary_materials_aalag199.zip › S4_QST.docx]

**Quantitative Sensory Testing**

NOTE: always perform testing in same order (TCR – MNT – TST – TNT). First side chosen at random.

**Environment**

- Horses are to be stabled at least 4h before testing to allow for acclimatization (ideally the night before).
- Testing is always performed in the same exam room. Try to minimize disturbances (lock door, keep floor quiet).
- Personnel present: examiner, groom for handling the horse, 1-2 helpers for recording of data/technical assistance.
- Room temperature is regulated and always maintained between 18-19°C.
- A companion horse (a horse of the clinic’s teaching herd or – if available – a horse from the same stable as the patient horse) is to be present for all tests.
- Horses are given at least a 30min break in their stall between TCR and QST. Add additional breaks if needed.

**Testing**

| Equipment: | - Algometer - *ProdPro, Topcat Metrology Ltd, Ely/UK* - stimulating tip 2mm - force increase 2 N/s, cutoff 25 N - Von Frey Filaments - *SENSELab Aesthesiometer, SOMEDIC Sales AB, Hörby/SE* - filament sizes 3-18; range 0.026-50g - Thermode device - *purpose-built handheld thermode device* - stimulating tip 1cm^2^ - temperature increase 0.5-0.6°C/s, cutoff 55°C |
| --- | --- |
|  |  |
| Testing: | - Apply device at respective site, perpendicular to skin (NOTE: MNT on unshaved, TNT on shaved/hairless areas) - Increase pressure/temperature, until horse shows a clear aversive reaction (i.e. head lifting/turning for MNT/TNT, blinking/nostril twitching for TST) - Repeat testing if difference between 1^st^ and 2^nd^ are MNT > 1 N / TNT > 3°C |
|  |  |

**Quantitative Sensory Testing**

| ID Sticker |  |  |  |
| --- | --- | --- | --- |
|  |  | Feasibility Score: |  |
|  |  | Room Temperature: |  |
|  |  | Skin Temperature: |  |
|  |  |  |  |

**Mechanical nociceptive threshold (MNT):**

| **Location** | **Force (N) that provoked reaction** | | | **Type of reaction** |
| --- | --- | --- | --- | --- |
|  | **1^st^** | **2^nd^** | **Repeat** |  |
| IOF – left/right |  |  |  |  |
| TMJ – |  |  |  |  |
| IOF – |  |  |  |  |
| TMJ – |  |  |  |  |

IOF – infraorbital foramen; TMJ – temporomandibular joint

**Tactile sensory threshold (TST): (Filaments used: 3, 6, 9, 12, 15, 18)**

| **Location** | **Filament size that provoked reaction** | **Type of reaction** |
| --- | --- | --- |
| Nostril – |  |  |
| LCE – |  |  |
| Nostril – |  |  |
| LCE – |  |  |

LCE – lateral cantus of the eye

**Thermal nociceptive threshold (TNT):**

| **Location** | **T° that provoked reaction** | | | **Type of reaction** |
| --- | --- | --- | --- | --- |
|  | **1^st^** | **2^nd^** | **Repeat** |  |
| IOF – |  |  |  |  |
| **T° increase** |  |  |  |  |
| MF – |  |  |  |  |
| **T° increase** |  |  |  |  |
| IOF – |  |  |  |  |
| **T° increase** |  |  |  |  |
| MF – |  |  |  |  |
| **T° increase** |  |  |  |  |

IOF – infraorbital foramen; MF – mental foramen
