# Supplementary material for: Diagnostic utility of trigeminocervical reflex and quantitative sensory testing in horses with trigeminal-mediated headshaking
Source: J Vet Intern Med. 2026 Sep 1;40(5):aalag199. doi: 10.1093/jvimsj/aalag199 (PMC13533209; doi:10.1093/jvimsj/aalag199)
Supplement: Supplementary file 1 [file supplementary_materials_aalag199.zip › S5_Signalment_HS.docx]

| Case | Age [y] | Breed | Sex | Use | Diagnosis | Duration of headshaking | Talbot Score^26^ | ES^27^ [%] |
| --- | --- | --- | --- | --- | --- | --- | --- | --- |
| 1 | 8 | Warmblood | Gelding | Leisure | TMHS | 6 mt | 3 | 67.9 |
| 2 | 6 | Freiberger | Mare | Leisure | TMHS | 1 mt | 2 | 35.7 |
| 3 | 13 | Warmblood | Mare | Showjumping | TMHS | 7 y | 2 | 32.1 |
| 4 | 7 | Warmblood | Gelding | Eventing | TMHS | 9 mt | 2 | 10.7 |
| 5 | 7 | Warmblood | Mare | Showjumping | TMHS | n.a. (> 1 y) | 1 | 10.7 |
| 6 | 17 | Warmblood | Mare | Leisure | Right-sided insertion desmopathy of nuchal ligament | n.a. (> 1 y) | 3 | 89.3 |
| 7 | 15 | Haflinger | Gelding | Driving | TMHS | 2 mt | 3 | 32.1 |
| 8 | 11 | Connemara | Mare | Leisure | TMHS | n.a. (> 1 y) | 2 | 32.1 |
| 9 | 12 | Warmblood | Gelding | Dressage | TMHS | 6 y | 2 | 0 |
| 10 | 15 | Warmblood | Gelding | Leisure | Cementoma 110 | 3 y | 2 | 35.7 |
| 11 | 18 | Warmblood | Gelding | Leisure | TMHS | 10 mt | 3 | 28.6 |
| 12 | 15 | Quarter Horse | Gelding | Leisure | TMHS | n.a. (> 1 y) | 3 | 17.8 |
| 13 | 15 | Warmblood | Gelding | Eventing | Fracture of left paracondylar process | n.a. (> 5 y) | 2 | 10.7 |
| 14 | 12 | French Pony | Mare | Leisure | TMHS | 13 mt | 3 | 21.4 |
| 15 | 9 | Warmblood | Gelding | Showjumping | TMHS | 18 mt | 3 | 57.1 |
| 16 | 8 | Warmblood | Gelding | Showjumping | TMHS | n.a. (> 1 y) | 3 | 50 |
| 17 | 25 | Warmblood | Mare | Leisure | Cementoma 407 | 1 mt | 3 | n.a. |
| Table S5: Signalment of horses presented for headshaking. TMHS = trigeminal-mediated headshaking; ES = Exercise Score | | | | | | | | |
