# Supplementary material for: Diagnostic utility of trigeminocervical reflex and quantitative sensory testing in horses with trigeminal-mediated headshaking
Source: J Vet Intern Med. 2026 Sep 1;40(5):aalag199. doi: 10.1093/jvimsj/aalag199 (PMC13533209; doi:10.1093/jvimsj/aalag199)
Supplement: Supplementary file 1 [file supplementary_materials_aalag199.zip › S6_Signalment_Control_Horses.docx]

| Case | Age [y] | Breed | Sex | Use/Origin |
| --- | --- | --- | --- | --- |
| 101 | 7 | Freiberger | Mare | Leisure / privately owned |
| 102 | 7 | Freiberger | Gelding | Leisure / privately owned |
| 103 | 17 | Freiberger | Gelding | Clinic |
| 104 | 16 | Warmblood | Gelding | Clinic |
| 105 | 12 | Warmblood | Gelding | Clinic |
| 106 | 16 | Warmblood | Gelding | Showjumping / privately owned |
| 107 | 11 | Warmblood | Mare | Clinic |
| 108 | 15 | Warmblood | Mare | Clinic |
| 109 | 14 | Warmblood | Mare | Clinic |
| 111 | 10 | Warmblood | Mare | Leisure / privately owned |
| 112 | 5 | Warmblood | Gelding | Leisure / privately owned |
| 113 | 8 | Haflinger | Mare | Leisure / privately owned |
| 114 | 17 | Connemara | Mare | Leisure / privately owned |
| 115 | 10 | Quarter Horse | Gelding | Western / privately owned |
| Table S6: Signalment of control horses. | | | | |
