# Supplementary material for: Diagnostic utility of trigeminocervical reflex and quantitative sensory testing in horses with trigeminal-mediated headshaking
Source: J Vet Intern Med. 2026 Sep 1;40(5):aalag199. doi: 10.1093/jvimsj/aalag199 (PMC13533209; doi:10.1093/jvimsj/aalag199)
Supplement: Supplementary file 1 [file supplementary_materials_aalag199.zip › S7_Results_sHS.docx]

|  | TCR threshold | | QST - MNT IOF | | QST - MNT TMJ | | QST - TST NOS | | QST - TST LCE | | QST - TNT IOF | | QST - TNT MF | |
| --- | --- | --- | --- | --- | --- | --- | --- | --- | --- | --- | --- | --- | --- | --- |
|  | *path* | *phys* | *path* | *phys* | *path* | *phys* | *path* | *phys* | *path* | *phys* | *path* | *phys* | *path* | *phys* |
| Horse 6 | 0.6 mA (R) | 2.1 mA (L) | 5.1 N | 6.2 N | 7.8 N | 4.5 N | 50 g | 110 g | 50 g | 50 g | 40.3°C | 43.3°C | 40.8°C | 49.1°C |
| Horse 10 | 0.7 mA (R) | 1.1 mA (L) | 3.1 N | 3.6 N | 3.9 N | 5.8 N | 0.026 g | 0.026 g | 0.026 g | 0.026 g | 47.3°C | 48.4°C | 42.2°C | 43.4°C |
| Horse 17 | 0.9 mA (R - IOF)  1.1 mA (R - MF) | 1.5 mA (L - IOF)  1.8 mA (L - MF) | 3.1 N | 1.1 N | 2.0 N | 3.1 N | 0.085 g | 0.085 g | 0.085 g | 0.085 g | 39.1°C | 41.6°C | 37.0°C | 46.3°C |
| TMHS | 0.82 mA |  | 5.4 N |  | 5.9 N |  | 28.33 g |  | 24.20 g |  | 44.6 °C |  | 46.2 °C |  |
| Controls |  | 1.16 mA |  | 6.4 N |  | 8.4 N |  | 7.22 g |  | 17.98 g |  | 44.2 °C |  | 47.3 °C |
| Table S7: Results of trigeminocervical reflex (TCR) and quantitative sensory testing (QST) for the horses with secondary headshaking.  path = pathological side; phys = physiological side; R = right; L = left; MNT = mechanical nociceptive threshold; TST = tactile sensory threshold; TNT = thermal nociceptive threshold; FS = feasibility score; IOF = infraorbital foramen; TMJ = temporomandibular joint; NOS = nostril; LCE = lateral cantus of the eye; MF = mental foramen | | | | | | | | | | | | | | |
