# Supplementary material for: Diagnostic utility of trigeminocervical reflex and quantitative sensory testing in horses with trigeminal-mediated headshaking
Source: J Vet Intern Med. 2026 Sep 1;40(5):aalag199. doi: 10.1093/jvimsj/aalag199 (PMC13533209; doi:10.1093/jvimsj/aalag199)
Supplement: Supplementary file 1 [file supplementary_materials_aalag199.zip › S8_ROC_QST.docx]

|  |  |
| --- | --- |
|  |  |
|  |  |

Figure S8: Receiver Operating Characteristic (ROC) Curves for different quantitative sensory testing modalities at two different sites each.
MNT = mechanical nociceptive threshold; TST = tactile sensory threshold; TNT = thermal nociceptive threshold; FS = feasibility score; IOF = infraorbital foramen; TMJ = temporomandibular joint; NOS = nostril; LCE = lateral cantus of the eye; MF = mental foramen
